# Ranked Sense Multiple Access Control Protocol for Multichannel Cognitive Radio-Based IoT Networks

**DOI:** 10.3390/s19071703

**Published:** 2019-04-10

**Authors:** Muhammad Shafiq, Pankaj Singh, Imran Ashraf, Maqbool Ahmad, Amjad Ali, Azeem Irshad, Muhammad Khalil Afzal, Jin-Ghoo Choi

**Affiliations:** 1 Department of Information and Communication Engineering, Yeungnam University, Gyeongsan 38541, Korea; shafiq.pu@gmail.com (M.S.); ashrafimran@live.com (I.A.); 2 Department of Electronics Engineering, Yeungnam University, Gyeongsan 38541, Korea; pankaj_singh86@ynu.ac.kr; 3 Department of Digital Convergence Business, Yeungnam University, Gyeongsan 38541, Korea; maqbool.pu@gmail.com; 4 Department of Computer Science, COMSATS University Islamabad, Lahore Campus, Lahore 54590, Pakistan; amjad.ali@cuilahore.edu.pk; 5 Department of Computer Science & Software Engineering, International Islamic University, Islamabad 44000, Pakistan; irshadazeem2@gmail.com; 6 Department of Computer Science, COMSATS University Islamabad, Wah Campus, Wah Cantt 47040, Pakistan; khalilafzal@ciitwah.edu.pk

**Keywords:** carrier sensing, CSMA/CA, IoT, rendezvous, spectrum sensing

## Abstract

The widespread growth of the Internet-of-Things (IoT) and its dependence on the license-exempt Industrial, Scientific, and Medical (ISM) bands have made spectrum resources scarce. IoT can nonetheless get advantage from the Cognitive Radio (CR) technology to resolve the spectrum shortage issue. Since in CR networks the unlicensed Secondary Users (SUs) can exploit the white spaces in licensed channels of Primary Users (PUs) opportunistically. CR ad hoc networks are more useful in IoT due to ease of installation, low cost, and less complexity. However, CR ad hoc networks are prone to the rendezvous issue and hidden primary terminal problem. Moreover, the available channels in the CR system are not identical, PUs’ and SUs’ activities can diversify them as well. In this connection, channel selection by SUs is a complex balancing act since the transmission opportunities are space, frequency and time bounded. In this paper, we hence proposed a new Ranked Sense Multiple Access with Collision Avoidance (RSMA/CA) protocol for multichannel CR-based IoT networks. Our proposed RSMA/CA protocol not only resolves the hidden primary terminal problem but also avoids hidden and exposed terminal problems at the same time by mutual spectrum sensing. We suggest a new channel ranking mechanism to rank the available channels based on the long term qualities of the channels, PUs’ return rate, and SUs’ activities and tailor-made the algorithms in an existing scheme to make the rendezvous process more efficient. We analyze the performance of our proposed RSMA/CA in terms of normalized throughput through the Markov chain model and compared with that of the existing scheme. Simulation results show that our RSMA/CA protocol outperforms the existing scheme due to efficient rendezvous and access mechanisms.

## 1. Introduction

**Figure 1:**
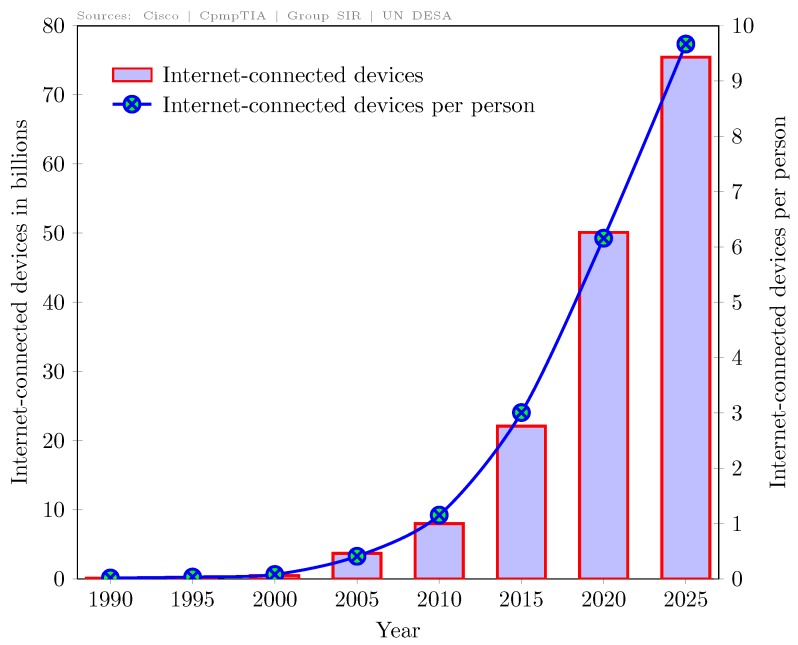
Internet-connected devices across the world.

In recent decades, Internet-of-Things (IoT) technology has drastically increased the number of devices connected to the Internet due to smart, low-cost, and low-power wireless objects like microcontrollers, sensors, actuators, etc. From human population prospects and some recent estimates [1,2,3], we have calculated that the count of Internet-connected devices will reach around 10 per person by 2025 if distributed evenly across the world as shown in Figure 1. This simply translates that the count of Internet-connected devices will be approximately 10 times more than the human population over this planet by 2025, which is more than 240% of 2015. The rapid growth of such IoT-enabled devices can revolutionize human lives in terms of the smart grid, smart cities, smart farming, connected health and much more [4,5]. However, it will require new communication technologies, purpose-built protocols and more spectrum otherwise. Furthermore, most of the IoT-enabling technologies such as WiFi, Bluetooth, Zigbee, RFID, etc. depend on the Industrial Scientific and Medical (ISM) bands for the Internet. Unfortunately, spectrum in ISM bands is limited. Therefore, it could not meet the demand for IoT devices in the near future.

The Cognitive Radio (CR) is considered as a promising technology that can resolve such a spectrum shortage issue. Therein, the unlicensed Secondary User (SUs) can adapt their operational parameters to exploit the white spaces in licensed channels [6,7,8,9]. The SUs can occupy the licensed channels whenever Primary User (PUs) are inactive in order to avoid the catastrophic interference. Once a PU returns, SUs must vacate the occupied channel within a tolerable delay [10]. This is so that a CR system is expected to be more than a conventional radio since it should accurately detect the transmission opportunities, learn from the environment, and take spontaneous actions to switch the licensed channels. One CR network can be deployed in a centralized or an ad hoc mode. CR ad hoc networks are more suitable to the IoT environment due to low cost, less complexity and ease of installation. However, such systems are prone to the hidden primary terminal problem due to decentralized environment, multipath fading and imperfect sensing [11,12,13].

In classical CSMA/CA protocol [14], a station identifies the state of the channel as idle or busy through the carrier sense mechanism before it enables collision avoidance and data transmission procedures. The carrier sensing includes sensing a channel by Clear Channel Assessment function to avoid the transmission overlap and the Network Allocation Vector (NAV) function to reserve the channel for data transmission. However, carrier sensing is limited because it is carried out at the high Signal-to-Noise-Ratio (SNR) regime, and so vulnerable to the hidden primary terminal problem. For example, if a PU is positioned outside the carrier sense range of a SU transmitter in a CR ad hoc network, it is hard to identify the activity status of this PU through the carrier sensing mechanism. In that case, SU transmitter can create harmful interference to the PU receiver. On the other hand, the spectrum sensing method such as energy detection, or cyclostationary feature detection mechanism [15,16,17] can be carried out in low SNR regime, but it requires significant time at the transmitter and receiver while introducing greater incidences of false alarm and misdetection.

In CR ad hoc networks, there exists a rendezvous problem among SUs as well. One popular approach in this connection is to use a dedicated out-of-band licensed channel, called Common Control Channel (CCC), so that SUs can coordinate each other to achieve rendezvous [18,19]. Nevertheless, CCC-based Medium Access Control (MAC) protocols theoretically look simple, but practically they are not much attractive due to the following drawbacks [20,21,22]. First, it is usually hard to identify a CCC throughout the network because of variations in time, space, and frequency. Second, the allocation cost and management issues of CCC have put forwarded the following key questions: “Who will offer CCC services?”; “How much the cost it would have?”; “What would be the payment method?” and “What happens if it is free?”, since a freely available CCC has a close resemblance to the unlicensed ISM bands. In that case, how to prevent such a free channel being used by the others for other than its sole purpose. Then, not only the traffic of other users can degrade the network performance, but also the appearance of PUs could measurably block the SUs’ access on CCC. Conversely, a dedicated CCC leased from the primary network is literally against the underlying objectives of the CR system. Third, the bandwidth limitation may lead to the early saturation of the control channel, and thus it could create bottleneck problem in the network. Finally, the channel security vulnerabilities and the single point of failure are the void open issues in this regard. For example, a simple jamming or Denial-of-Service attack could disrupt the functionality of the entire network.

The Channel Hopping (CH) mechanisms otherwise are of interest since they do not require CCC to achieve rendezvous [23,24,25]. The CH method is inspired by the Adaptive Frequency Hopping (AFH) of Bluetooth technology [26], where the Bluetooth nodes in piconet hop channels following a CH sequence generated by the master device. The AFH is simple in design yet not feasible in CR-based IoT networks due to their large scale environment. However, CH is adopted in the CR system with some variations into two major categories called sequence-based CH and random CH. These mechanisms are sufficiently immune to the vulnerabilities that the CCC approach suffers. In sequence-based CH, each SU transmitter visits (or hops) all the available channels through a predefined hopping pattern until it established a communication link with the intended SU receiver [23]. On contrary to this, each SU under the random CH visits network channels in a random pattern at a certain hopping rate. In such CH schemes, the total time is first divided into various time slots of fixed duration. Then, the rendezvous process is started where each SU tries to pair with its counterpart at a common available channel. Towards this end, we henceforth termed the number of slots used by a pair of SUs in the discovery of a common channel as Time to Rendezvous (TTR).

The random CH cannot guarantee that two SUs will successfully establish a rendezvous because its TTR is not upper-bounded. However, sequence-based CH has the advantage of finite TTR. It can further be evaluated under synchronous and asynchronous approaches in terms of synchronization of SUs. Synchronous CH approaches assume global clock synchronization in secondary network. In that case, a pair of SUs can start CH simultaneously and when their CH sequences overlap, rendezvous process is achieved successfully. On the other hand, asynchronous CH approaches are supposed to be the candidate solution that does not require global clock synchronization. Both synchronous and asynchronous schemes can be based on either symmetric or asymmetric channel paradigms. In the symmetric channel paradigm, a pair of indented SUs have the same number of available channels, while SUs may have a different number of available channels in the asymmetric channel paradigm. In CR ad hoc networks, the number of available channels may not be similar for SUs due to their different locations. Therefore, this can make the rendezvous process more complex.

In multichannel CR networks, a pair of SUs may have multiple available channels that the rendezvous algorithm can identify during the MAC operation. The intermediate question is how to select the channel, and how to carry out data transmission once a channel is selected. At the same time, PUs can autonomously adopt a variety of applications (e.g., voice, video, or data) with different traffic patterns [27]. Therefore, under the uneven traffic pattern and unpredictable behavior of PUs, channel selection becomes a complex balancing act. For best channel selection, a CR may learn PUs’ and SUs’ traffic by maintaining a channel history, which can make a MAC protocol more efficient. In literature, channel selection is mostly done based on the current information of the network channels. The available channels could be assumed as equally good [28,29] or evaluated by bandwidth [30,31] or categorized by the least interference level [32,33]. A CR randomly detects transmission opportunities by spectrum sensing or/and carrier sensing and can select a channel at a moment that is otherwise frequently used by the PUs or/and SUs. Moreover, if the underlying scheme (e.g., bandwidth-based selection) is already in use by PUs, SUs may select a bad channel. Therefore, to maintain seamless transmissions in secondary network, it is desirable to adopt a ranked channel selection by MAC protocol, which should be based on the prior knowledge of the users’ traffic.

In this paper, we design a new MAC protocol, called Ranked Sense Multiple Access with Collision Avoidance (RSMA/CA), for multichannel CR-based IoT networks. The RSMA/CA is aimed to establish rendezvous between the pair of SUs by allocating one of the best channels based on the ranked selection without relying on the dedicated CCC. We tailor made the existing algorithms in BRACER [34] to resolve the rendezvous problem in a fast time, in which a transmitter and its corresponding receiver individually construct their CH sequences in a beacon period first. Then, transmitter follows its CH sequence to hop and sense the ranked channels by carrier sensing. If a hopped channel is found idle for Data Interframe Space (DIFS) interval, it broadcasts a Beacon to Sense (BTS) packet to its corresponding receiver to achieve rendezvous. On the other hand, the receiver stays on ranked channels according to its own CH sequence. Therein, if the receiver decodes the BTS packet correctly, it then replies with the Acknowledge to Sense (ATS) packet to the transmitter and ensures rendezvous. Once the rendezvous is ensured, the transmitter broadcasts a Request to Sense (RTS) packet to its receiver in order to continue the mutual spectrum sensing in a data period. In the sequel, transmitter and its receiver conduct mutual spectrum sensing at the same time. If no PU is active around the transmitter and the receiver, then data transmission continues on the chosen ranked channel when receiver replies with the Clear to Sense (CTS) packet. Otherwise, SUs are blocked by spectrum sensing for a predefined period. The receiver replies with the ACK packet when it correctly receives the data packet. In case of collision, SUs nevertheless follow the backoff process as part in classical CSMA/CA protocol. We rank the channels based on the long term qualities as well as the SUs’ activities and PUs’ return rate to reduce interference and channel handoffs.

The key contribution of this paper is summarized as follows.

- We propose a simple but immanent MAC protocol for multichannel channel CR-based IoT networks, which not only resolves the intrinsic hidden primary terminal problem but also fixes the classical hidden and exposed terminal problems at the same time.
- We suggest algorithms for the construction of hopping sequences in secondary transmitter and receiver by modifying the exiting BRACER protocol in the minimal to achieve rendezvous in an efficient time.
- We propose a novel ranking mechanism to downsize the available channels, which is based on the past information of the channels’ qualities as well as the activities of SUs and PUs enabling IoT devices selecting the best channels to reduce rendezvous time and plummet PUs’ interference and SUs’ collisions on those channels.
- We analyze the normalized throughput of our proposed protocol with the Markov chain model and verify through the Monte Carlo simulations in the MATLAB. We also compared the throughput of our proposed MAC with that of the exiting BRACER protocol.

For the quick reference, we provide a summary of the acronyms in Table 1. The rest of this paper is structured as follows. In Section 2, we present an overview of the related works. In Section 3, we define the system model. In Section 4, we describe the proposed MAC protocol. In Section 5, we investigate its performance and compared with the existing scheme in terms of normalized throughput. In Section 6, we discuss the simulation results. Finally, we conclude the paper in Section 7.

## 2. Related Works

Here, we give a brief overview of the related multichannel CR MAC protocols with the state-of-the-art channel selection algorithms adopted in the existing literature.

In [23], an asynchronous Sequence-Based Rendezvous (SBR) scheme is proposed, which is based on the permutations of *C* available channels. However, its average TTR is a quadratic function of *C*. It requires higher access delay especially when the number of network channels is large. In [35], two algorithms, called synchronous efficient channel hopping and asynchronous efficient channel hopping schemes, are proposed. Both algorithms build CH sequences to work in the symmetric channel mechanism in a way similar to that in SBR scheme. This is how they are less attractive for multichannel CR ad hoc networks under asymmetric channel conditions. In [19], three algorithms called Modular Clock (MC), Generated Orthogonal Sequence (GOS), and Modified Modular Clock (MMC) are developed. MC and GOS work in the symmetric channel environment for a pair of SUs to generate predefined and random CH sequences, respectively. On the other hand, MMC is proposed for the asynchronous channel environment. However, all these three algorithms do not guarantee rendezvous in a finite time.

In [36], a jump-stay based asymmetric mechanism is proposed that overcomes the drawbacks of the MC algorithm. This algorithm generates CH sequences with two different patterns, called jump and stay, each. In the jump pattern, a SU jumps (or hops) on *C* available channels equal to the prime number (P) time slots, where P is larger than *C*. In the stay pattern, it stays on a particular channel for 2P time slots to achieve rendezvous. The jump-stay algorithm guarantees rendezvous. However, its average TTR has polynomial complexity with respect to *C* available channels. In [37], an asynchronous CH algorithm is proposed, where each SU require a unique *n*-bit pattern as an identification sequence. For that purpose, MAC address can be considered which can inflate the TTR due to 48-bit long identifier.

In [38], a Quorum-based CH (QCH) system is introduced with two asynchronous algorithms to achieve rendezvous by the intersection property of quorums. This system requires a priori information of all SUs over the available channels, which often obtained through the network broadcasts with high power consumptions in SUs. Therefore, the feasibility of QCH system in the power contained networks is limited. In [34], BRACER protocol is proposed to avoid broadcast collisions and achieve rendezvous in multichannel CR ad hoc networks. In BRACER, all SUs downsize the available channels in a way such that at least one common channel exists between the HSs of SUs. This scheme introduces two asynchronous algorithms to construct CH sequences in asymmetric channel paradigm and guarantee rendezvous. However, still there exists ample research room in multichannel CR ad hoc networks to reduce delay in the rendezvous operation.

In [39], a decentralized MAC protocol is proposed, where PU traffic behavior is modeled based on the Partially Observable Markov Decision Process (POMDP). In POMDP, a reward-based suboptimal greedy approach is adopted by the SUs for the best channel selection. However, the transitional probabilities assumed in the Markov channel model with the advance knowledge of PUs’ slots makes this protocol unrealistic. In [40], a slotted MAC protocol, called SYN-MAC, without CCC is proposed. In SYN-MAC, SUs are assumed to be equipped with two different radios. One radio is dedicated for listening to the control signal in a time slot, which is assigned to each of the available channels. Contrariwise, the other radio is reserved for serving the data traffic. To achieve synchronization, a SU transmits beacon messages separately on the available channels at the beginning of time slots. Similarly, the rest of the SUs tune their control radios on each of the available channels and exchange the available channel sets in response to the beacon messages received periodically. After synchronization, when a secondary transmitter wants to transmit a data packet to the specified secondary receiver, it blindly finds a common channel and exchanges control messages. After the successful sharing of control messages, data transmission starts. Although SYN-MAC works without CCC but its common channel selection from the available channel sets is prone to be reclaimed by the PU. In worse condition, PU is highly likely to reclaim the licensed channel just after its selection is made by the SU. Consequently, it will degrade the overall network performance.

## 3. System Model

We consider one decentralized secondary network with *M* SUs and multiple primary networks with *C* licensed channels. SUs can coexist with PUs yet cannot collaborate with each other. However, SUs can collaborate with each other through a peer-to-peer communication setup [41]. SUs can occupy one of the licensed channels whenever PUs on that channel are inactive. However, SUs are obliged to vacate the channel when PUs are active. A transmitter SU *i* and its receiver SU *j* can sense their adjacent PUs as active or inactive by one of the spectrum sensing techniques such as energy detection, cyclostationary feature detection, and so on [15,16,17].

The spectrum sensing at SU *i* cannot detect the activity status of the adjacent PUs of SU *j* due to its limited range. Hence, SU *j* conducts spectrum sensing to detect the activity status of its adjacent PUs in conjunction with SU *i*, which we called mutual spectrum sensing. In this sense, we called the adjacent PUs of SU *j* as the hidden PUs of SU *i*. However, spectrum sensing is not perfect due to false alarm and misdetection. We denote the false alarm probability and the misdetection probability of SU *i* as $\alpha_{i}$ and $\beta_{i}$, respectively. False alarm implies when a SU detects an inactive PU as active due to sensing error. The false alarm probability by which a SU *i* lose an opportunity can be found in [15] as,

(1) $$\alpha_{i}=Q\left(\left(\frac{\omega }{\upsilon^{2}}-1\right)\sqrt{\delta f_{s}}\right),$$

where Q(·) denote the complementary distribution function of a standard Gaussian variable. ω, υ, δ, and $f_{s}$ represent the sensing threshold, noise power, sensing time, and sampling rate of the rendezvous-channel, respectively. However, misdetection happens when a SU identifies the active PUs as inactive due to sensing error. The misdetection probability by which a SU *i* creates interference is calculated in [15] as,

(2) $$\beta_{i}=1-Q\left(\frac{1}{\sqrt{2\gamma +1}}Q^{-1}\left(\alpha_{i}\right)-\sqrt{\delta f_{s}}\gamma \right),$$

where γ denotes SNR of the primary signal measured at SU *i*. The misdetection probability should be limited by a threshold ${\hat{\beta }}_{i}$ such that $\beta_{i}\leq {\hat{\beta }}_{i}$ to sufficiently protect the PUs.

## 4. Proposed Protocol

Here, we discuss the operation of our RSMA/CA protocol, which consists of the following three phases. Before we proceed, we provide a summary of the used symbols in Table 2.

### 4.1. Channel Negotiation

In this subsection, we discuss how a transmitter SU *i* conducts channel negotiation with a receiver SU *j* during the rendezvous operation. We consider a duty cycle approach, in which a transmitter and its corresponding receiver periodically hop the available channels based on the hopping sequences. The hopping sequences are the sequences of available channels by which a transmitter and its receiver hop for the successful rendezvous in a common channel. However, channel hopping overhead could be increased measurably if the number of available channels is large. Hence, SUs only hop the downsized ranked channels in order to reduce the switching overhead. In this connection, we devise the ranking process of the downsized channels as detailed in Section 4.2.

**Figure 2:**
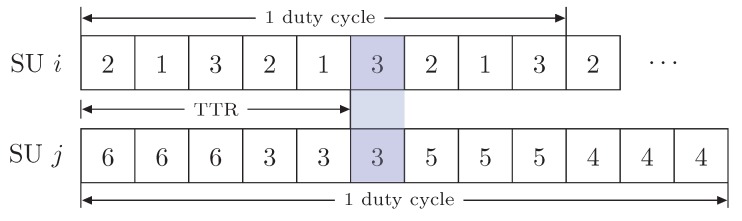
Illustration of the hopping sequences at SUs *i* and *j*.

For the rendezvous operation, the transmitter SU *i* and its receiver SU *j* individually construct their hopping sequences according to Algorithms 1 and 2, respectively. Our Algorithms are similar to that in BRACER [34] yet with the tailor-made feature of the efficient rendezvous. We describe Algorithm 1 as follows. First, the transmitter SU *i* randomizes the downsized ranked channel set $X_{i}$ to pick an edge at a moment. SU *i* initializes its hopping sequence $HS_{i}$ to be empty and variable *k* to be 1 (lines 1–3). Then, SU *i* constructs its hopping sequence $HS_{i}$ to intermittently traverse the downsized ranked channels for ${\left(Y_{i}\right)}^{2}$ time slots per duty cycle (lines 4–8). On the other hand, we explain Algorithm 2 as follows. First, the receiver SU *j* randomizes downsized ranked channel set $X_{j}$ and initializes its hopping sequences $HS_{j}$ to be empty and variable *k* to be 1 (lines 1–3). Then, SU *j* constructs hopping sequence $HS_{j}$ using the nearest prime number P less than its downsized ranked channel set size $Y_{j}$ (lines 4–12). Note that the same procedure is repeated for each downsized ranked channel. Following Algorithm 2, the receiver SU *j* stays on each ranked channel for at least $Y_{j}-1$ time slots per duty cycle. We aimed to reduce the receiver’s stay on the ranked channels in order to make the rendezvous process faster. For that purpose, we use the largest prime such that $P<Y_{j}$ (line 7 of Algorithm 2) that enables SU *j*’s stay on each of the ranked channels in more efficient time, which is less than that of BRACER [34]. We consider an example of the hopping sequences for the transmitter SU *i* and its corresponding receiver SU *j* in Figure 2, so as to explain the rendezvous operation. The downsized available channel set of SU *i* is {1,2,3} and that of SU *j* is {3,4,5,6} with one common available channel. Therefore, the size of downsized ranked channels at SUs *i* and *j* is 3 and 4, respectively. Following Algorithm 1, SU *i* generates its hopping sequence $HS_{i}$ as {2,1,3,2,1,3,2,1,3} having $3^{2}\left(=9\right)$ time slots. Similarly, SU *j* constructs its hopping sequence $HS_{j}$ as {6,6,6,3,3,3,5,5,5,4,4,4} according to Algorithm 2. The SU *j* is staying for at least 4−1(=3) time slots on each of the ranked channels per duty cycle. This can be observed that SUs *i* and *j* established their first rendezvous in time slot 6 at channel 3. Thus, TTR by SU *i* to establish a link with SU *j* commensurate to the time duration of 6 slots.

In a rendezvous process, SU *i* first hops following its own hopping sequence and then transmits a control packet called Beacon to Sense (BTS) on each downsized ranked channel to establish a link with its receiver SU *j*. On the other hand, SU *j* hops according to its own hopping sequence and so listen on each downsized ranked channel. If SU *j* overhears the BTS packet, it can then respond with another control packet called Acknowledge to Sense (ATS) on an arbitrary channel, which we called the rendezvous-channel. The exchange of BTS and ATS packets on the rendezvous-channel ensures successful rendezvous. However, SUs *i* and *j* can guarantee rendezvous if there exists at least one common channel in their hoping sequences. So, the rendezvous time of SU *i* can be calculated as,

(3) $$TTR=\sum_{k=1}^{e}BTS{}_{k}+ATS,$$

where *e* accounts for the number of channels traversed by SU *i* to find SU *j* until the rendezvous-channel *e*. Furthermore, BTS and ATS denote transmission time of BTS and ATS packets, respectively.

**Algorithm 1** Construction of hopping sequence $HS_{i}$ for rendezvous operation of a SU transmitter *i*.**Input:**$X_{i}$, $Y_{i}$;
**Output:**
$HS_{i}$
 1: Randomize the order of $X_{i}$; 2: $HS_{i}\leftarrow \emptyset$; 3. k←1; 4: **while**
$k\leq {\left(Y_{i}\right)}^{2}$
**do** 5:  $HS_{i}\left(k\right)\leftarrow X_{i}\left(k\;\mathrm{rem}\;Y_{i}+1\right)$; 6:  k←k+1; 7: **end while** 8: **return**
$HS_{i}$;

**Algorithm 2** Construction of hopping sequence $HS_{j}$ for rendezvous operation of a SU receiver *j*.**Input:**$X_{j}$, $Y_{j}$, P;
**Output:**
$HS_{j}$
 1: Randomize the order of $X_{j}$; 2: $HS_{j}\leftarrow \emptyset$; 3: k←1; 4: **while**
$k\leq Y_{j}$
**do** 5:  l←1; 6:  **while**
$l\leq Y_{j}$
**do** 7:   $HS_{j}\left(\left(k-1\right)P+l\right)\leftarrow X_{j}\left(k\right)$; 8:   l←l+1; 9:  **end while** 10:  k←k+1; 11: **end while** 12: **return**
$HS_{j}$;

### 4.2. Channel Selection

We adopt a ranking mechanism for the reliable channel selection, which is based on the past information of PUs and SUs as well as the long term peculiarities of available channels. The past information (e.g., channel status, SNR, allowed power, users’ activities, etc.) can help in the selection of best channel to reduce interference and switching overhead. Following a predictive approach, it is always hard to exactly predict the future activities of the users. However, a CR can gain sufficient intelligence based on such information while ranking the channels. In our scheme, each SU stores sensing results in a database table, which includes the activity status of PU (denoted by $A^{c}$ ) with that of SU (denoted by $\Lambda^{c}$) and SNR of channel *c* through its past observations in spectrum sensing and carrier sensing as illustrated in Table 3. SU can consider past *t* sensing periods to compute channel quality $q^{c}$ at t+1-th period of channel *c*. At period *t*, our system takes sensing results $\left\{E_{i}^{c}\left(t\right)\right\}$ for SU *i* as the input and outputs the channel rank $R^{c}\left(t;q^{c}\right)$ as well as the long term channel rank ${\tilde{R}}^{c}\left(t;q^{c}\right)$ of a channel *c*. We define the quality of a channel as follows,

(4) $$q^{c}=\left(A^{c}+\Lambda^{c}\right)\gamma^{c},$$

where we recall that $\gamma^{c}$ represents the SNR of channel *c*. We set the $A^{c}\left(=\Lambda^{c}\right)=1$ when a PU (or SU) is found inactive and otherwise it will be 0. Therefore, the channel quality is marked as high when the activity status of PU ($A^{c}$) and/or that of SU ($\Lambda^{c})$ is equal to 1 for a given SNR ($\gamma^{c}$) since the state of the channel is the dominant factor. If the state of the two different channels is the same in terms of PUs and SUs activities, SNR will then become the dominant factor to determine the channel quality. On the other hand, we compute the long term rank of a channel with the following iteration,

(5) $${\tilde{R}}^{c}\left(t;q^{c}\right)=\left(1-\epsilon \left(t\right)\right){\tilde{R}}^{c}\left(t-1;q^{c}\right)+\epsilon \left(t\right)R^{c}\left(t;q^{c}\right),$$

where ϵ(t) is the weight factor chosen such that ϵ(t)>0, $\lim_{t\to \infty }\epsilon \left(t\right)=0$, and $\sum_{t}\epsilon \left(t\right)\to \infty$ [42]. The long term average rank of a channel (${\tilde{R}}^{c}\left(t;q^{c}\right)$) converges for a small weight factor (ϵ(t)).

We can then denote the available channel with the highest long term average rank as,

(6) $$c^{*}=\underset{c\in \{1,\ldots ,C\}}{arg max}{\tilde{R}}^{c}\left(t;q^{c}\right),$$

where *C* is the number of available channels. Finally, we set the channel quality of the next sensing period t+1 in order to compute the rank of the chosen channel as,

(7) $$R^{c}\left(t+1\right)=q^{c^{*}}.$$

If ${\tilde{R}}^{c}\left(t;q^{c}\right)$ converges for every *c*, the optimal index $c^{*}$ of Equation (6) also converges. As a result, the rank $R^{c}\left(t+1\right)$ follows that of the $c^{*}$th of the chosen channel according to Equation (7). In each turn, SUs can similarly evaluate ranks of the remaining channels by choosing the highest *Y* ranked channel one by one. SU *i* and *j* can now downsize the available channels, in which k∈{1,⋯,C} highest ranked channels can be chosen for the rendezvous process. The value of *Y* should be chosen in a way such that there exists at least one common channel between the downsized ranked channel sets $X_{i}$ and $X_{j}$ at SU *i* and SU *j* to guarantee rendezvous. In this regard, the detailed method can be found in [34]. The downsized channels should be limited by a threshold not only to avoid unnecessary rendezvous delay but also to conduct transmission on a high-quality channel.

### 4.3. Channel Sharing

**Figure 3:**
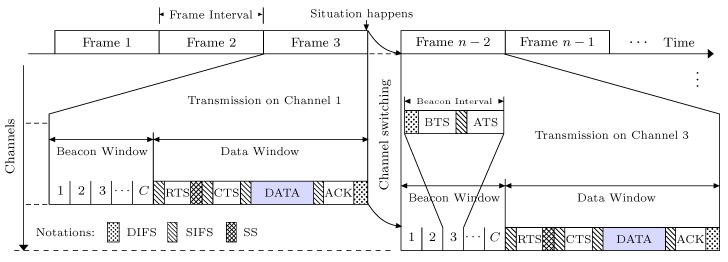
RSMA/CA access mechanism in time channel domain. BTS, Beacon to Sense; ATS, Acknowledge to Sense; RTS, Request to Sense; CTS, Clear to Sense; SS, Spectrum Sensing.

In this subsection, we discuss the channel sharing process of our proposed RSMA/CA protocol, which splits the time scale into non-overlapping frames as illustrated in Figure 3. Each frame consisting of a beacon window and a data window, respectively. In the beacon window, SUs hop the ranked channels in different time slots, in which a SU transmitter first performs carrier sensing during a DIFS interval to ensure the silence of other SUs. When the channel is clear, the transmitter exchanges the BTS and ATS packets to achieve rendezvous. However, the data window begins once a rendezvous is achieved between a pair of SUs in an arbitrary slot of the beacon window. In the data window, SUs follow the collision and interference avoidance mechanism by enabling a standard backoff procedure and a mutual spectrum sensing, respectively.

The transmitter whose backoff expired first broadcasts the Request to Sense (RTS) packet to its corresponding receiver and then conducts spectrum sensing to ensure the silence of adjacent PUs. Conversely, SU receiver overhears the Clear to Sense (CTS) packet and conducts spectrum sensing as well to ensure the silence of hidden PUs. The SU receiver replies with the CTS packet if the hidden PUs are found inactive and otherwise it holds the CTS packet to avoid interference. Similarly, SU transmitter stops the transmission if the adjacent PUs are found active. Therefore, the successful sharing of RTS and CTS packets not only ensures the silence of adjacent (and hidden) PUs but also holds the other SUs from accessing the rendezvous-channel through NAV updating. In the sequel, SU transmitter sends data packet and thereafter SU receiver replies with the ACK packet to complete a transmission. SUs can otherwise switch to another channel if the rendezvous-channel is blocked by incumbent PUs. The detailed operation under RSMA/CA protocol is outlined in Section 5.

## 5. Performance Analysis

We evaluate the performance of our RSMA/CA protocol in the following in terms of normalized throughput.

### 5.1. Packet Transmission Process

In this subsection, we analyze the packet transmission process under the proposed RSMA/CA protocol. Each SU with a packet to send initiates data transmission wherever its backoff counter reaches to 0 at rendezvous-channel. In once packet transmission attempt, each SU can encounter one the following four events: (1) Blocking at transmitter, (2) RTS packets collision, (3) Blocking at receiver, and (4) Successful transmission, which are denoted as $e_{1}$, $e_{2}$, $e_{3}$, and $e_{4}$, respectively. We henceforth calculate the occurrence probability (*P*) and time delay (*T*) for each of the four possible events. In this connection, we define the idle channel probability $\pi_{i}$ of a SU *i* such that the rendezvous-channel is sensed free from the activity of incumbent PUs during the spectrum sensing as,

(8) $$\pi_{i}={\left(\left(1-\alpha_{i}\right)V_{0,i}+\beta_{i}V_{1,i}\right)}^{K},$$

where *K* is the number of common channels in the hopping sequences of SUs *i* and *j*; $\alpha_{i}$ and $\beta_{i}$ denote the probability of false alarm and that of misdetection for SU *i*, respectively. Therefore, when SU *i* conducts spectrum sensing, it decides the status of the rendezvous-channel as idle with probability $\pi_{i}$ and as blocked with blocking probability $b_{i}=1-\pi_{i}$.

In our RSMA/CA protocol, we recall that SUs follow standard backoff process to avoid collisions. For one packet transmission, each transmitter first chooses its backoff counter at random, which is decreased by one with each idle backoff slot. The transmitter whose backoff counter first reaches 0 can transmit the RTS packet. Suppose that one transmitter SU *i* has the backoff counter 0 with backlogged buffer queue and it has achieved rendezvous to its receiver SU *j* at an arbitrary channel. Thus, SU *i* transmits RTS packet to SU *j* with a transmission trial probability $\theta_{i}$. SU *i* then conducts spectrum sensing and so observes event $e_{1}$, when it sensed the adjacent PUs as active, with probability,

(9) $$P_{e_{1}}=1-\pi_{i}.$$

**Figure 4:**
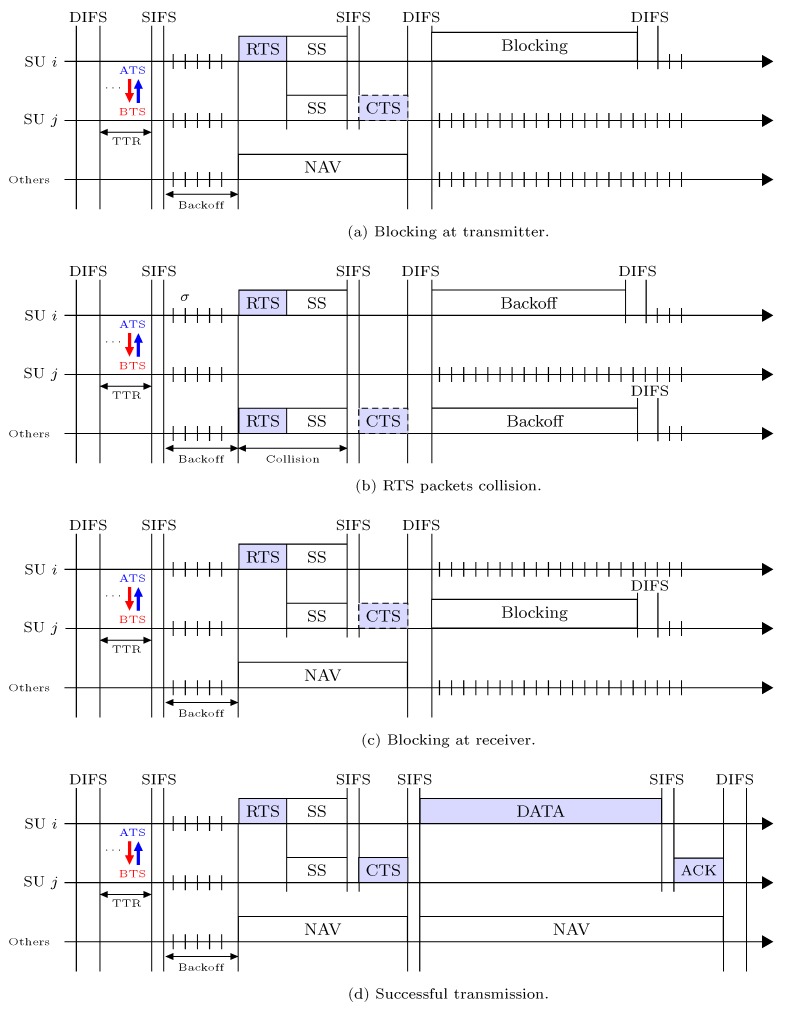
RSMA/CA access mechanism in the rendezvous-channel followed by the all possible events: (**a**) event $e_{1}$; (**b**) event $e_{2}$; (**c**) event $e_{3}$; (**d**) event $e_{4}$.

Figure 4a shows time delay that a SU *i* spends due to event $e_{1}$, i.e., the duration of $e_{1}$ slot, which can be written as,

(10) $$T_{e_{1}}=TTR+RTS+SS+2SIFS+CTS+DIFS,$$

where RTS and CTS represent the transmission time of one RTS packet and that of one CTS packet, respectively. Further, SS accounts for the duration of a spectrum sensing.

Suppose that SU *i* has transmitted the RTS packet to SU *j* and during the spectrum sensing it founds the rendezvous-channel as idle. However, SU *i* becomes failed to receive the CTS packet (from the SU *j*) because its RTS packet has collided with the RTS packets of other SUs (their backoff counters are expired with that of the transmitter SU *i* at the same time). We called that situation as event $e_{2}$, which happens with probability,

(11) $$P_{e_{2}}=\pi_{i}\left(1-\prod_{n\neq i}\left(1-\pi_{n}\theta_{n}\right)\right),$$

where $\pi_{n}$ and $\theta_{n}$ respectively represent rendezvous-channel idle probability and transmission trial probability of SU *n*. In Equation (11), this could be helpful to note that RTS of SU *i* collides with the RTS packet of at least one SU out of *n* users, when n≠i, where n∈[1,M], are not trying to transmit at backoff counter 0 with probability $\prod_{n\neq i}\left(1-\pi_{n}\theta_{n}\right)$. As shown in Figure 4b, the time delay a SU *i* spends at the rendezvous channel due to event $e_{2}$ can be given as,

(12) $$T_{e_{2}}=TTR+RTS+SS+2SIFS+CTS+DIFS.$$

Suppose that SU *i* has transmitted the RTS packet and is waiting to receive the CTS packet from the SU *j* because it founds the rendezvous-channel as idle. However, spectrum sensing can block the SU *j* for a predefined duration if its adjacent PUs (or SU *i*’s hidden PUs) are found active. In that situation, SU *j* cannot return CTS packet to SU *i*. Eventually, SU *i* gives up transmission after time-outs in order to protect the hidden PUs, which is event $e_{3}$ that occurs with probability,

(13) $$P_{e_{3}}=\pi_{i}b_{j}\sum_{i\neq j}\tau_{ij}\left(\prod_{n\neq i,j}\left(1-\pi_{n}\theta_{n}\right)\right),$$

where $b_{j}$ denotes rendezvous-channel blocking probability for SU *j*, and $\tau_{ij}$ accounts for SU *i*’s data packets transmission probability to SU *j*. Equation (13) indicates that SU *i* does not collide with any of the *n* users, where n≠i,j, such that n∈[1,M], are not trying to transmit. Figure 4c shows the time delay of SU *i* due to event $e_{3}$, which can be written as,

(14) $$T_{e_{3}}=TTR+RTS+SS+2SIFS+CTS+DIFS.$$

For the successful transmission case, suppose that SUs *i* and *j* have successfully exchanged the RTS and CTS packets such that they are not blocked in the mutual spectrum sensing. Thereafter, SU *i* transmits a data packet to SU *j*. Therefore, thanks to the NAV mechanism, SU *i* receives the ACK packet from its receiver SU *j* without interruption on an error-free rendezvous-channel. We called that situation as event $e_{4}$, which occurs with the probability as follows,

(15) $$P_{e_{4}}=\pi_{i}\pi_{j}\left(1-\theta_{j}\right)\sum_{i\neq j}\tau_{ij}\left(\prod_{n\neq i,j}\left(1-\pi_{n}\theta_{n}\right)\right),$$

where $1-\theta_{j}$ denotes that SU *j* is in the reception sate. Figure 4d shows the time delay of SU *i* in a successful data packet transmission due to event $e_{4}$, which can be written as,

(16) $$\begin{array}{cc} T_{e_{4}} & =TTR+RTS+SS+4SIFS\\ & +CTS+DATA+ACK+DIFS, \end{array}$$

where DATA and ACK represent the transmission time of a DATA packet and that of an ACK packet, respectively.

### 5.2. Normalized Throughput

In this subsection, we analyze the normalized throughput of the proposed RSMA/CA protocol with the following assumptions:

- The topology of the secondary network is a fully connected graph, in which SUs are distributed with a single hop distance and are directly connected to each other.
- The secondary network is in the saturated state such that each SU always has backlogged queue with at least one DATA packet to send.
- The size of the downsized ranked channel sets have at least one common available channel at the transmitter SU *i* and its corresponding receiver SU *j*.
- The ranking of the downsized channels remains stable for one successive transmission period.
- There is no capture effect in the rendezvous-channel because it is error-free. Thus, a packet is only dropped when there is a collision in the system.
- The control and DATA packets are transmitted at the same rate through the rendezvous-channel, which is shared among all SUs.
- The physical layer, PU return rate, and transmission rate are the same and constant for all SUs.

Our RSMA/CA adopts the Binary Exponential Backoff (the collision avoidance algorithm of the IEEE 802.11 standard) process to avoid collisions. Therein, a SU *i* begins backoff with the initial contention window size, $W_{0}$, from the backoff stage 0. Thus, the initial backoff counter, *u*, is chosen between $\left(0,W_{0}-1\right)$ and decreases 1 with every idle backoff slot. The SU starts packet transmission when the backoff counter becomes 0. If that transmission is successful, the backoff stage becomes 0 and it moves up to k+1 otherwise when there is a collision. In case of collision, the backoff counter is chosen between $\left(0;W_{k+1}\right)$. At the final stage *N*, SU *i* can continue to attempt without any limit with the final window size $W_{N}$ until the packet is transmitted.

**Figure 5:**
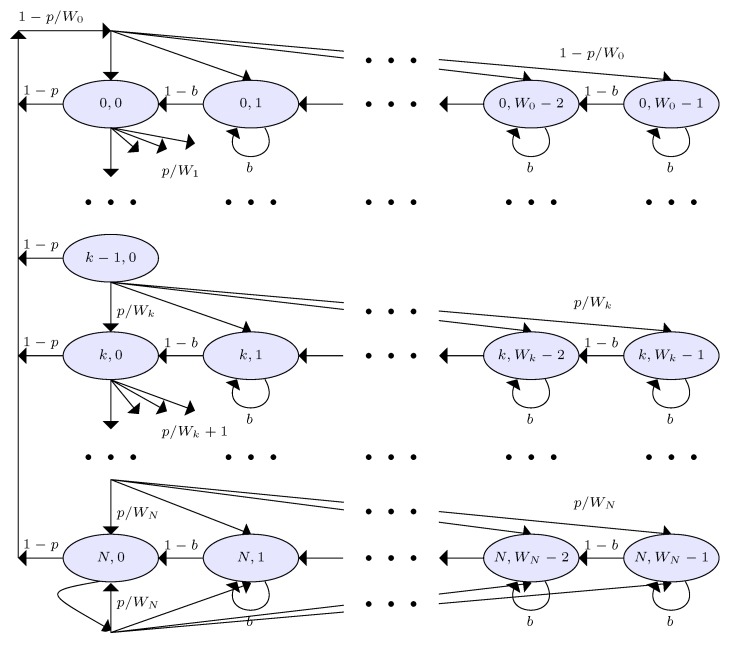
Markov model for the backoff process in RSMA/CA.

We henceforth dropped the subscript *i* without loss of generality. We model the backoff process of our proposed RSMA/CA protocol with the two dimensional Markov model as illustrated in Figure 5. In this model, backoff state (k,u), is defined as state of a SU *i* at an arbitrary time *t* being the values of two stochastic processes of backoff stage k(t) and backoff counter u(t), where k∈[0;N] and $u\in [0;W_{k}]$ in the stage *k*, respectively. If the PU is found active, SU *i* freezes its backoff counter for a blocking period with blocking probability *b* and releases its backoff counter with clearance probability 1−b in the backoff stage k(≥0). SU *i* can either observe a failed transmission or a successful transmission in one packet transmission attempt at backoff stage 0. We denote the failed transmission probability of SU *i* with *p*. From Equations (9), (11) and (13), we can see that,

(17) $$p=P_{e_{1}}+P_{e_{2}}+P_{e_{3}}.$$

Hence, SU *i* can observe a failed transmission with probability *p* due to events $e_{1}$, $e_{2}$, and $e_{3}$ and it can get a successful transmission due to event $e_{4}$ with probability 1−p, i.e., $=P_{e_{4}}$ as Equation (15) since $P_{e_{1}}+P_{e_{2}}+P_{e_{3}}+P_{e_{4}}=1$. We notice that the Markov model of our RSMA/CA protocol is similar to that in [43] due to the homogeneous backoff procedure. From the results in [43], we hence refer to the the state transition probabilities and the transmission trial probability of SU *i* directly as Equations (18) and (19).

(18) $$\left\{\begin{array}{cc} \mathrm{Pr}\{(k,u)\to (k,u)\}=b\; & \mathrm{for}\;k\in \left[0,N\right],\;u\in \left[1,W_{k}-1\right)\\ \mathrm{Pr}\{(k,u-1)\to (k,u)\}=1-b\; & \mathrm{for}\;k\in \left[0,N\right],\;u\in \left[1,W_{k}-1\right)\\ \mathrm{Pr}\left\{\left(0,u\right)\to \left(k,0\right)\right\}=\frac{1-p}{W_{0}-1}\; & \mathrm{for}\;k\in \left[0,N-1\right],\;u\in \left[1,W_{k}-1\right)\\ \mathrm{Pr}\left\{\left(k,u\right)\to \left(k-1,0\right)\right\}=\frac{p}{W_{k}-1}\;\; & \mathrm{for}\;k\in \left[1,N\right],\;u\in \left[1,W_{k}-1\right)\\ \mathrm{Pr}\left\{\left(0,u\right)\to \left(N,0\right)\right\}=\frac{1}{W_{N}-1}\;\; & \mathrm{for}\;u\in [1,W_{k}-1). \end{array}\right.$$

(19) $$\theta_{i}=\sum_{k=0}^{N}\left(k,0\right)=\frac{1-p^{N+1}}{1-p}\left(\frac{2(1-b)(1-2p)(1-p)}{W\left(1-{\left(2p\right)}^{N+1}\right)\left(1-p\right)+2\left(1-2p\right)\left(1-p^{N+1}\right)\left(1-b\right)}\right).$$

**Figure 6:**
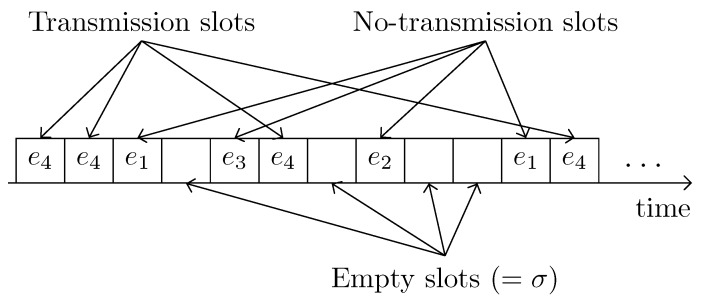
Classification of the system slots in RSMA/CA.

We can distinguish the system slots into the empty slots, transmission slots, and no-transmission slots as illustrated in Figure 6. The empty slots occur when SUs remain in the backoff states and so they do not attempt to transmit packets. The occurrence probability of all those empty slots in the proposed system can radially be obtained as,

(20) $$P_{e}=\prod_{n}\left(1-\theta_{n}\right).$$

We know that SUs can observe the successful transmissions whenever event $e_{4}$ happens. We called all those slots as the transmission slots that occur with probability,

(21) $$P_{t}=\sum_{n}\theta_{n}\left(1-p_{n}\right),$$

where $1-p_{n}$ accounts for successful transmission probability of SU *n*, where n∈[1,M]. However, all those slots that contain failed transmissions (due to $e_{1}$, $e_{2}$, or $e_{3}$) correspond to the no-transmission slots. The occurrence probability of such slots can be obtained as $P_{n}=1-\left(P_{t}+P_{e}\right)$.

We now can express the normalized throughput of our proposed RSMA/CA system as the ratio,

(22) $$S=\frac{\mathrm{Average}\;\mathrm{data}\;\mathrm{bits}\;\mathrm{transmitted}\;\mathrm{in}\;a\;\mathrm{system}\;\mathrm{slot}}{\mathrm{Average}\;\mathrm{length}\;\mathrm{of}\;a\;\mathrm{system}\;\mathrm{slot}}.$$

Let E[D] be an average Payload-Data-Unit (PDU) size in bits of an autonomous system. From Equation (21), the average PDU bits successfully transmitted in one system slot time is given as $P_{t}E\left[D\right]$. Let E[L] be the average length of a system slot time that can radially be obtained as,

(23) $$E\left[L\right]=P_{e}\sigma +P_{t}E\left[T_{t}\right]+P_{n}E\left[T_{n}\right],$$

where $T_{t}=T_{4}$ and $T_{n}=T_{2}\left(=T_{1}=T_{3}\right)$. From Equation (16), we can compute the average $T_{t}$ as follows,

(24) $$\begin{array}{cc} E[T_{t}] & =TTR+RTS+SS+4SIFS\\ & +CTS+DATA+ACK+DIFS,\\ & =E[TTR]+RTS+SS+4SIFS\\ & +CTS+(H+E[D])/R+ACK+DIFS, \end{array}$$

where *H*, and *R* denote the total size of MAC and PHY headers, and the transmission rate of rendezvous-channel respectively. However, E[TTR] represents the average time to rendezvous, which is given by,

(25) $$E\left[TTR\right]=\frac{\sum_{n}\theta_{n}\left(1/{\left(TTR_{n}\right)}^{K}\right)}{E[L]},$$

since a successful rendezvous has negative correlation to *K*, which is the number of common channels in the hopping sequences of SUs. In Equation (25), this could be helpful to notice that $\theta_{n}(1/{\left(TTR_{n}\right)}^{K}$ is the successful rendezvous probability of SU *n*. From Equation (14), we can see that $E\left[T_{n}\right]=T_{2}$, since $T_{2}$ is constant. Finally, Equation (22) can be written as,

(26) $$S=\frac{P_{t}E\left[D\right]}{P_{e}\sigma +P_{t}E\left[T_{t}\right]+P_{n}E\left[T_{n}\right]},$$

which is the overall normalized throughput of our system.

## 6. Results and Discussion

In this section, we investigate the performance of our proposed RSMA/CA protocol and compare it with BRACER of [34]. The performance results are verified by the Monte Carlo simulations, which are developed in the MATLAB with 10,000 runs. We consider the number of available channels as 20. The transmission rate is set equal for each channel. However, the channel quality varies in terms of SNR, SU activities and PU return rate according to the uniform distribution. For each channel, the SNR value is chosen from [−25, 25], the number of SUs is chosen from [2, 20], and PU return rate is chosen from [0.01, 0.25]. In a nutshell, the default simulation parameters are summarized in Table 4.

**Figure 7:**
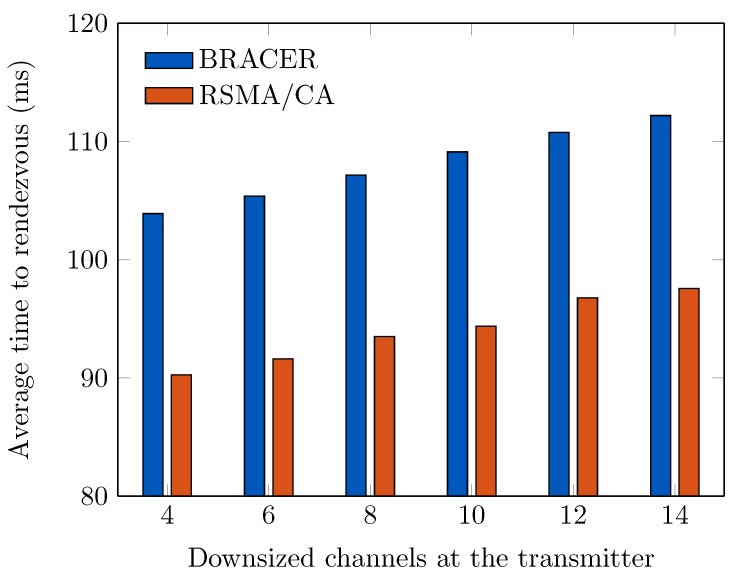
Average TTR vs. *Y* at the Tx (on Y=15 at the Rx).

In Figure 7, we show the average TTR where the size of downsized channels *Y* at the transmitter varies from 2 to 14 on the fixed *Y* (which is equal to 15) at the receiver. We witness that the average TTR increases steadily as the size of the downsized channel increases. This is so that a large *Y* can yield large rendezvous overhead at the transmitter. We also witness that our RSMA/CA protocol outperforms BRACER due to its less rendezvous delay. Since the receiver in our RSMA/CA scheme stays for fewer slots on the ranked channels compared to that in BRACER protocol.

**Figure 8:**
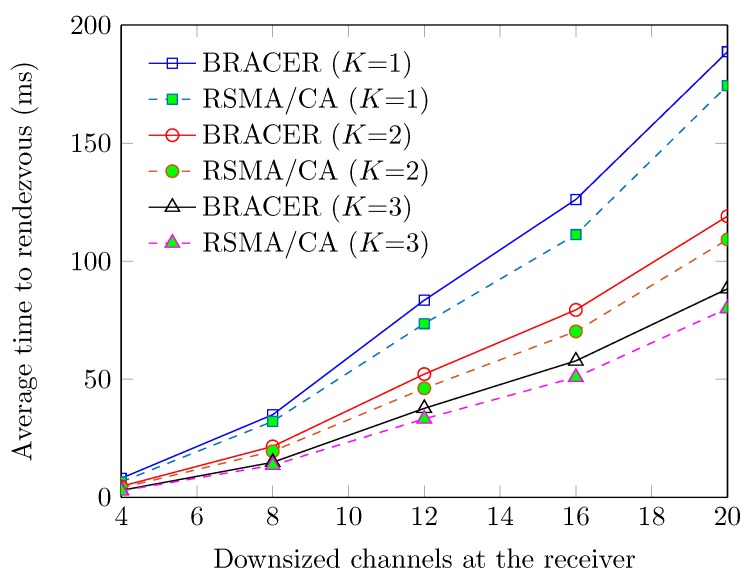
Average TTR vs. *Y* at the receiver (for various *K*s).

In Figure 8, we also demonstrate how average TTR is affected on the fixed size of downsized channels (with Y=2) at the transmitter when *Y* at the receiver varies from 2 to 14 for various *K*s in HSs of transmitter and receiver. We see that the average TTR monotonically increases with the increase in *Y* at the receiver due to the increased number of time slots in the duty cycle of the transmitter. Conversely, the value of average TTR decreases with the increase in common channels *K* in HSs of the transmitters and their corresponding receivers. This is because as long as the values of *K* increased, chances of rendezvous are increased and so the rendezvous is achieved successfully in a less delay. We also observe that the proposed RSMA/CA protocol outperforms BRACER due to fast rendezvous between SUs.

**Figure 9:**
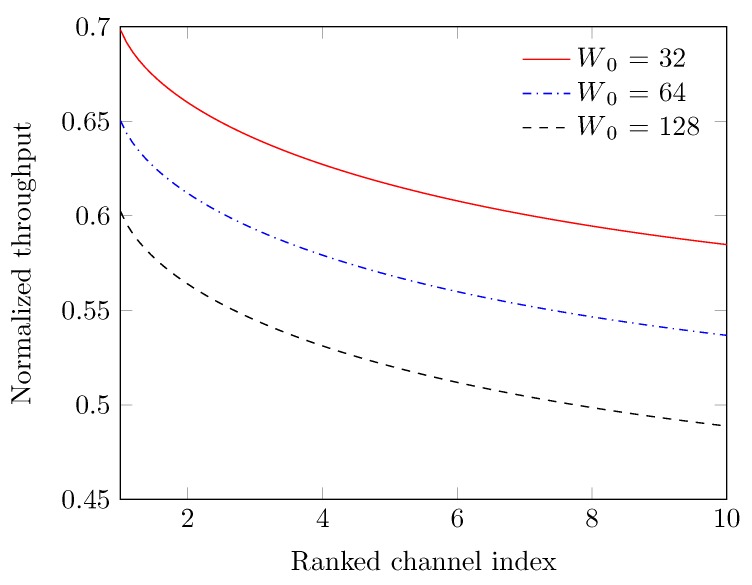
Normalized throughput vs. ranked channel index.

We provide the normalized throughput of the different ranked channels for various contention windows in Figure 9, where we consider the ranked channel $R^{c}$ in highest to lowest order at index c=1,⋯,10, respectively. We observe that normalized throughput decreases with the increase in the ranked channel index. This is because of the fact that channels are ranked based on the SUs’ traffic, PUs’ return rate, and channel quality in terms of SNR. Therefore, the highest ranked channel produces better throughput due to less blocking rate, better quality, and limited collisions among SUs. However, the gaps between the curves of normalized throughput are attributed due to various sizes of the contention window $W_{0}$. When the $W_{0}$ is large, the rendezvous-channel time is being wasted by the backoff slots, because backoff counter chosen by SUs on average becomes large for the default number of SUs. The system has then low normalized throughput. In addition, if the $W_{0}$ is small, the backoff counter chosen by SUs on average is enough to avoid collisions among the fewer SUs. Then, the system has a high normalized throughput.

**Figure 10:**
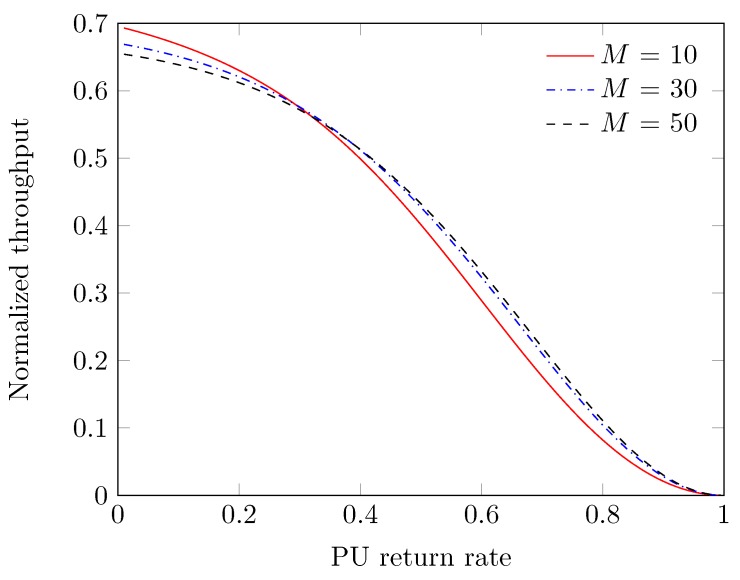
Normalized throughput vs. PU return rate.

In Figure 10, we show the effect of PU return rate over the system throughput for the various number of SUs. We see that the normalized throughput monotonically decreases with the increase in PU return rate since the rendezvous-channel access probability decreases. When the PU return rate is low, the achieved normalized throughput is better for fewer SUs *M* due to fewer collisions. On contrary to this, if the PU return rate is high, the achieved normalized throughput is then better for the large *M*. Since the large *M* can produce more packets compared to the small *M*. However, the overall throughput of the system decreases due to a large number of SUs’ blocking at the transmitter and receiver.

**Figure 11:**
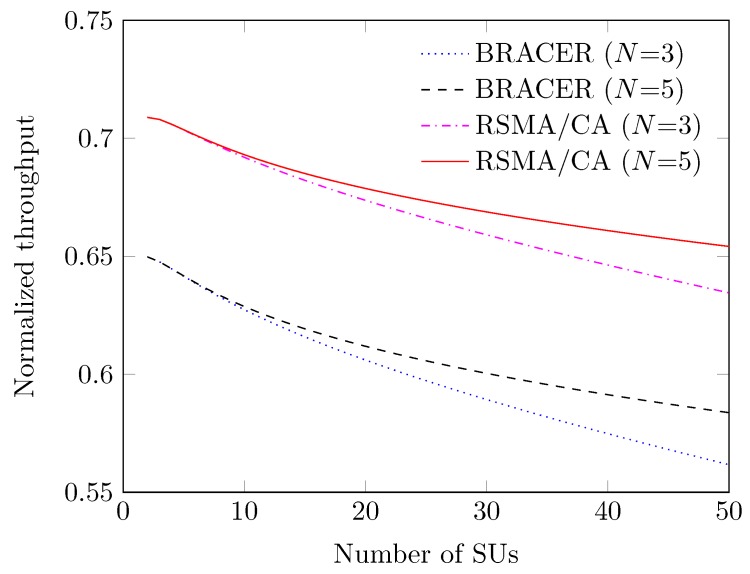
Normalized throughput vs. number of SUs.

We investigate the throughput of our RSMA/CA protocol over the number of SUs and compared with that of BRACER under the system of interest in Figure 11, where we consider the maximum backoff stage *N* of 3 and 5, respectively. The throughput decreases as the number of SUs increases since the collision rate among SUs increases. We observe that the normalized throughput increases when *N* is large even with more collisions because the backoff counter chosen is large, and it decreases otherwise. We also witness that our RSMA/CA protocol outperforms BRACER in terms of normalized throughput due to its efficient rendezvous and access mechanisms.

## 7. Conclusions

In this paper, we have proposed a new MAC protocol, called RSMA/CA, for multichannel CR-based IoT networks. Our scheme resolves the hidden primary terminal problem as well as the classical hidden and exposed terminal problems at the same time. In our RSMA/CA, each station first identifies the state of the ranked channels as idle or busy through the carrier sensing and then enables the BTS/CTS mechanism to achieve rendezvous. If a channel is sensed idle, it then conducts a random access mechanism with mutual spectrum sensing technique. To this end, the heterogeneous nature of radio spectrum precludes ranking of the non-identical channels since the sensing information of one specific channel may not be a useful indicator for the ranking of another channel. Furthermore, ranking the channels based on the information abstracted from the current activity of the spectrum sensing on all those channels measurably increased the rendezvous delay. Therefore, we rank the available channels through the more recent long term observations of spectrum sensing and carrier sensing for stable transmissions. The available channels are chosen based on their ranks to reduce the rendezvous delay. We have investigated the performance of proposed RSMA/CA protocol in terms of normalized throughput using the Markov model and compared with the previous scheme. Simulation results reveal that our RSMA/CA is a good candidate MAC protocol for multichannel CR networks since its performance is insensitive to the number of available channels.

## Figures and Tables

**Table 1:** Summary of the acronyms.

Acronym	Definition
ACK	Acknowledgement
AFH	Adaptive Frequency Hopping
ATS	Acknowledge to Sense
BTS	Beacon to Sense
CCC	Common Control Channel
CH	Channel Hopping
CR	Cognitive Radio
CTS	Clear to Sense
DIFS	Data Interframe Space
GOS	Generated Orthogonal Sequence
IoT	Internet-of-Things
ISM	Industrial, Scientific, and Medical
MC	Modular Clock
MMC	Modified Modular Clock
NAV	Network Allocation Vector
PDU	Payload-Data-Unit
PHY	Physical layer
POMDP	Partially Observable Markov Decision Process
QCH	Quorum-based CH
RSMA/CA	Ranked Sense Multiple Access with Collision Avoidance
RTS	Request to Sense
SBR	Sequence-Based Rendezvous
SIFS	Short Interframe Space
SNR	Signal-to-Noise-Ratio
SS	Spectrum Sensing
SUs	Secondary Users
TTR	Time to Rendezvous

**Table 2:** Summary of the symbols.

Symbol	Description
$HS_{i}$	Hopping sequence for a transmitter SU *i*
$HS_{j}$	Hopping sequence for a receiver SU *j*
*X*	Downsized ranked channel set of an arbitrary SU
*Y*	Size of the downsized ranked channel set of an arbitrary SU
P	Largest prime number such that P<Y
$V_{1,i}$,	Active probability of PUs positioned around SU *i*
$V_{0,i}$	Inactive probability of PUs positioned around SU *i*
$\alpha_{i}$	False alarm probability of sensor in SU *i*
$\beta_{i}$	Misdetection probability of sensor in SU *i*
$\pi_{i}$	Idle probability of rendezvous-channel for SU *i*
$P_{e_{i}}$	Probability of event $e_{i}$ (where i=1,2,3,4) at arbitrary time
$T_{e_{i}}$	Time delay of event $e_{i}$ (where i=1,2,3,4)
*R*	Transmission rate of rendezvous-channel
*p*	Failed transmission probability
*b*	Blocking probability of rendezvous-channel
*K*	Common channels in hopping sequences of SUs *i* and *j*
*C*	Number of available channels in CR system
*M*	Maximum number of SUs in the system
*e*	Rendezvous-channel of SUs *i* and *j* at arbitrary time
*H*	Header size of PHY and MAC
σ	Length of one backoff (or idle) slot
S	Normalized throughput of RSMA/CA
$\theta_{i}$	Transmission trial probability for SU *i*
*k*	Backoff stage of an arbitrary SU
$W_{k}$	Size of contention window at *k*-th stage
*N*	Backoff process of an arbitrary SU at final stage
$W_{0}$	Initial size of contention window
$W_{N}$	Final size of contention window
E[L]	Average length of one system slot
$E_{i}^{c}\left(t\right)$	Sensing result of SU *i* at channel *c* and period *t*
$\tau_{i,j}$	Packet transmission probability of SU *i* to SU *j*
$P_{t}$	Occurrence probability of a transmission slot
$P_{n}$	Occurrence probability of a no-transmission slot
$P_{e}$	Occurrence probability of an empty slot
*D*	Average size of one packet payload for an arbitrary SU

**Table 3:** Illustration of the sensing table in SU *i*. Legend: 1, Inactive; 0, Active.

Time	$A^{1}$	$\Lambda^{1}$	${\mathrm{SNR}}^{1}$	$A^{2}$	$\Lambda^{2}$	${\mathrm{SNR}}^{2}$	…	…	…	…	…	…	$A^{C}$	$\Lambda^{C}$	${\mathrm{SNR}}^{C}$
$E_{i}^{c}\left(1\right)$	1	0	25	1	-	-	0	1	20	…	…	…	1	1	25
$E_{i}^{c}\left(2\right)$	0	0	28	0	-	-	1	0	23	…	…	…	1	0	34
$E_{i}^{c}\left(3\right)$	0	1	10	1	-	-	0	0	10	…	…	…	0	0	17
⋮	⋮	⋮	⋮	⋮	⋮	⋮	⋮	⋮	⋮	⋮	⋮	⋮	⋮	⋮	⋮
$E_{i}^{c}\left(t-1\right)$	1	1	27	0	-	-	0	0	25	…	…	…	1	0	32
$E_{i}^{c}\left(t\right)$	0	0	10	1	-	-	1	1	−7	…	…	…	0	0	−5

**Table 4:** Default parameters used in simulations.

Parameter Name	Value
PHY header	120 bits
MAC header	272 bits
PDU size	8184 bits
BTS/RTS	160 bits + PHY header
ATS/CTS/ACK	112 bits + PHY header
Number of common channels (*K*)	1
Downsized channel set size *Y* in Tx (Rx)	2(3)
Misdetection probability threshold ($\hat{\beta }$)	0.1
SIFS interval	10 μs
DIFS interval	50 μs
Backoff slot interval (σ)	20 μs
Spectrum sensing interval	0.5 ms
Final backoff stage (*N*)	5
Initial contention window size ($W_{0}$)	32
Final contention window size ($W_{N}$)	1024
Rendezvous-channel transmission rate (*R*)	1 Mbps
